# Analyzing facial action units in children to differentiate genuine and fake pain during inferior alveolar nerve block: a cross-sectional study

**DOI:** 10.1038/s41598-023-42982-6

**Published:** 2023-09-20

**Authors:** Muaaz Alkhouli, Zuhair Al-Nerabieah, Mayssoon Dashash

**Affiliations:** Faculty of Dentistry, Damascus University, Damascus, Syrian Arab Republic

**Keywords:** Human behaviour, Medical research, Paediatric research

## Abstract

This study aimed to investigate the association between facial action units and pain levels in Syrian children, focusing on both genuine and fake pain expressions. A total of 300 Syrian children aged 6–9 years participated in the study. Pain levels were assessed using the validated Face, Legs, Activity, Cry, Consolability scale, and facial expressions were analyzed using the Facial Action Coding System. The children were asked to mimic their feelings after receiving a dental injection to elicit fake pain expressions. Statistical analysis, including multinomial logistic regression and chi-square tests, was conducted to determine the Action Units (AUs) associated with each pain level and to compare the differences between real and fake pain expressions. The results revealed significant associations between specific AUs and pain levels. For real pain expressions, the most activated AUs across different pain levels with positive coefficient values of correlation (*P*-value < 0.01) were analyzed. In contrast, for fake pain expressions, AU12 and AU38 were consistently observed to be the most activated. These findings suggest that certain AUs are uniquely associated with fake pain expressions, distinct from those observed in real pain expressions. Furthermore, there were no significant differences between boys and girls in terms of their genuine and fake pain expressions, indicating a similar pattern of AU activation (*P*-value > 0.05). It was concluded that AUs 4, 6, 41, and 46 were associated with mild pain, and AUs 4, 6, 41, 46, and 11 were associated with moderate pain cases. In severe pain, AUs 4, 6, 7, 9, 11, and 43 were associated. In fake pain feelings, AU43, AU38, and AU12 were the most activated with no difference between boys and girls.

## Introduction

In 2020, the International Association for the Study of Pain (IASP) defined pain as “An unpleasant sensory and emotional experience associated with, or resembling that associated with, actual or potential tissue damage^1^”. However, pain is not a general perception. It is always a personal experience, which is affected, to varying degrees, by biological, racial, psychological, and social factors^2^. In children, the expression of pain is more complex^3^. The motor, social and psychological development of the child are some factors that make children's pain fingerprints that could not be generalized^4^.

Facial expressions play a crucial role in understanding and analyzing pain, as they serve as a nonverbal communication channel for individuals to express their subjective experiences^5^. The intricate and subtle changes in facial muscle movements can provide valuable information about the presence of pain, its intensity, and nature^6^. Examining facial expressions during painful experiences enables healthcare professionals and researchers to better comprehend the subjective experiences of individuals, allowing for more accurate pain assessment and tailored pain management strategies^7^.

Pain management during dental procedures is of utmost importance, particularly in the field of pediatric dentistry, where fear and anxiety may be heightened among young patients^8^. One common source of discomfort in dental settings is the administration of local anesthesia through injections^9^. Dental injections can elicit pain and distress, potentially leading to negative experiences and subsequent dental anxiety^10^. This will negatively affect the children's attitude and parental satisfaction^11^. Therefore, understanding the facial expressions associated with dental injection pain can aid in the development of effective pain management strategies and improve the overall dental experience for children.

The Facial Action Coding System (FACS), a comprehensive tool for analyzing facial expressions, provides a systematic framework to identify and quantify specific facial muscle movements known as action units (AUs)^12^. By examining the presence and intensity of these AUs, researchers can identify patterns associated with pain and distress^13^. The child Facial Coding System (CFCS) is a modified version of the FACS, which has been validated in children for the assessment of pain^14,15^.

Fake pain expressions refer to intentional or simulated facial movements aimed at mimicking pain without experiencing the actual sensation^16^. The majority of research has focused on the analysis of genuine or authentic pain expressions. There is also a need to understand the characteristics and patterns of fake pain expressions^17^.

Studying fake AUs provides valuable insights into the ability of individuals to consciously control their facial expressions and the potential implications for pain assessment^18^. The examination of fake AUs is particularly relevant in various clinical settings, including the assessment of pain in individuals who may attempt to conceal or exaggerate their discomfort, such as children or patients with communication difficulties^17,19^.

A recent systematic review (2019) concluded that there is a schism of research findings about the specific AUs associated with pain^20^. There is also a scarcity of research investigating the specific AUs associated with dental injection pain in Syrian children. For that reason, this study aimed to address this research gap by determining the AUs commonly observed in the facial expressions of Syrian children undergoing dental injections in the hope to enable pain assessment and management in pediatric dentistry.

Moreover, there is limited research focusing on the analysis of fake pain expressions and their association with specific AUs^20^. Therefore, present study also aimed to identify the specific AUs that are commonly activated in fake pain expressions.Kindly check and confirm the section headings are correctly identified.they were correctly identified 

## Methods

### Study design

The current study was an observational, cross-sectional study designed in line with the COnsensus-based Standards for the selection of health Measurement INstruments (COSMIN) statement for 9 months from May 2022 to February 2023. It was approved by the Research Ethics Committee of the Faculty of Dentistry at Damascus University on September 22, 2021, with approval number ‘3662’. In addition, all experiments were performed in accordance with relevant guidelines and regulations.

### Participants

The study involved children between 6 and 9 years old who required an inferior alveolar nerve block (IANB) for dental procedures. A total of 300 children were recruited from the Pediatric Dentistry Department at Damascus University. Informed consent was obtained from the parents of the participants and it included a clear explanation of the objectives of the study. Children with a history of chronic pain conditions, neurological disorders affecting facial expressions, cognitive impairments, or allergic reactions to amide anesthetics were excluded from the study.

### Sample size

In this study, the sample size was calculated according to a formula developed by Kothari in 2004. $\mathrm{n}=\frac{P\left(1-P\right)}{\frac{{A}^{2}}{{Z}^{2}} + \frac{P\left(1-P\right)}{N}/R}$

N The estimated population size was 3000 patients treated in the Department of pediatric dentistry at Damascus University within the last three months.

P is the estimated variance for the population, which was considered as 50%.

A is the desired precision, which was set at 0.05.

z was 1.96 as the confidence level was set at 95%.

R is the response rate of the audience and it was set as 80% to account for potential dropouts.

The calculated sample size was 286 children. However, the sample size was increased to 300 children to ensure adequate representation.

### Intervention procedure

Before the dental procedures, the children were seated comfortably in a dental chair in a well-lit examination room. They were instructed to sit still and keep their faces visible to the cameras throughout the procedure.

Two high-resolution cameras (iPhone 13 Pro Max) were positioned strategically to ensure optimal recording of facial expressions and body movements. One camera was mounted on a mobile holder, which was stabilized on the dental chair arm to capture a frontal view of the child's face, while the other camera was positioned at a side angle to capture any body movements that are required for the Face, Legs, Activity, Cry, Consolability (FLACC) scale. The cameras were positioned at a suitable distance to ensure clear visibility of the child's facial expressions and minimize any interference with the dental procedure.

Once the cameras were set up, a trained pedodontist performed the dental procedures, including the administration of the inferior alveolar nerve block. The dentist followed standard protocols for the administration of local anesthesia, ensuring the child’s safety and comfort throughout the procedure. Half of the 1.8 ml ampule of Lidocaine with epinephrine (1:100,000) was injected using a conventional syringe with a 30-gauge needle. Before that, 20% Benzocaine gel (Darby Dental Supply, NY) was applied to the insertion point of the needle and was allowed to take effect for 1 min to minimize any discomfort.

### Pain assessment

Pain assessment was performed using the Face, Legs, Activity, Cry, Consolability (FLACC) scale. The entire dental procedure, including the administration of the inferior alveolar nerve block, was recorded using two cameras. The video recordings captured both the dentist's actions and the child's facial expressions and body movements during the procedure (especially during the needle insertion).

Two trained examiners, who independently scored the FLACC scale based on the children's behavior, during injection, reviewed the video recordings. The examiners carefully observed the recorded videos, paying close attention to the child's facial movements, body movements, crying, and other behavioral indicators of pain. The FLACC scale is a 10-point pain assessment tool. It was divided into four grades of pain: zero (no pain), 1–3 (mild pain), 4–6 (moderate pain), and 7–10 (severe pain). Inter-examiner reliability between the two examiners was assessed using Cohen's kappa. The test showed a high level of agreement between the two examiners (kappa = 0.89), indicating a reliable scoring process. In addition, the intra-examiner reliability test was conducted by repeating the scoring process for 10% of the sample by each rater after a period of 10 days, which showed an excellent level of agreement with a kappa value of 0.89 and 0.92, respectively.

### Facial action units (FAUs) coding

Two additional examiners (M.A and Z.A), who were trained in the coding of facial expressions using the Child Facial Coding System (CFCS), analyzed the video recordings. The recorded facial videos were transferred from the cameras to a computer for analysis. The videos were organized and labeled with unique identifiers for each participant to ensure data integrity and confidentiality. To analyze the FAUs, frames or still images were extracted from the videos at specific time intervals or key moments during the dental injection. These frames were selected based on the visibility and clarity of the child's facial expressions. The extracted frames were saved as Joint Photographic Experts Group (JPEG) image files for subsequent FAU analysis. Adobe Photoshop software was used to display and analyze the extracted frames. This software allowed the raters to view and manipulate the images and zoom in on specific facial regions to identify the presence or absence of specific FAUs.

A total of 22 FAUs were analyzed (14 upper face AUs and 8 lower-face AUs). All obscured FAUs, which were associated with lips and mouth and may not be clear during the anesthesia, were excluded. For example, AU 13 (cheek puffer), AU 18 (lip puckerer), AU 20 (lip stretcher), AU 23 (lip tightener), AU 24 (lip pressor) and AU 28 (lip suck) were not analyzed in our study. This was carried out to minimize the confounding factors (injections), predominantly involve the oral and perioral regions, including the lips and mouth. These lower face AUs are closely associated with mouth closing and they cannot be obvious while performing the anesthesia. Table 1 shows the studied FAUs with their facial movements. To evaluate the reliability of FAUs coding, a subset of the images was randomly selected for re-coding by each examiner (M.A and Z.A). The test showed an excellent level of agreement with a kappa value of 0.88 and 0.89, respectively. Moreover, the tests showed a high level of agreement between the two raters, with a kappa value of 0.85.

**Table 1:** The studied FAUs with their facial movements.

Upper face AUs		Lower face AUs	
AU	FACS action	AU	FACS action
1	Inner brow raiser	9	Nose wrinkle
2	Outer brow raiser	10	Upper lip raiser
4	Brow lowerer	11	Nasolabial deepener
5	Upper lid raiser	15	Lip corner depressor
6	Cheek raiser	16	Lower lip depressor
7	Lid tightener	17	Chin raiser
41	Lid droop	26	Jaw drop
42	Slit	12	Lip corner puller
43	Eyes closed		
44	Squint		
45	Blink		
46	Wink		
38	Nostril dilator		
39	Nostril compressor		

In the second phase, participants were asked to mimic or simulate their feelings of pain after completing the dental injection. This involved intentionally replicating facial expressions associated with pain. Similar to the real FAUs analysis, the participants' facial expressions were recorded using. The recorded videos were later coded for AUs using the same coding system as in the genuine pain.

### Statistical analysis

Descriptive data, including frequency and percentages of FAUs in both real and fake pain, were summarized. The statistical analysis was performed using the IBM SPSS software v. 23 (IBM Corp., Armonk, USA). Multinomial logistic regression was conducted to study the association of both real and fake FAUs with pain levels. The level of significance (*P*-value) was set at 0.01%. The goodness-of-fit of the multinomial logistic regression model was assessed using the likelihood ratio and the Hosmer–Lemeshow test. Additionally, the chi-square test was used to study the difference between boys and girls. The level of significance (*P*-value) and power of the study were set at 0.05% and 90%, respectively.

## Results

Facial videos of 300 Syrian children (135 boys and 165 girls) receiving IANB were analyzed to determine the FAUs that were activated. The mean age of the participants was 7.5 years (SD = 0.7). The frequencies and percentages of pain levels assessed by the FLACC scale are summarized in Table 2.

**Table 2:** Frequencies and percentages of pain levels assessed by the FLACC scale.

	Level 0	Level 1	Level 2	Level 3
N* (%)	62 (20.7%)	102 (34%)	88 (29.3%)	48 (16%)
Boys	30	58	29	18
Girls	32	44	59	30

*Number of children.

## FAUs (14 upper face AUs and 8 lower face AUs) were analyzed in this study. Table 3 shows the frequencies of activation of each AU in each pain level.

**Table 3:** Frequencies of activation of each AU in each genuine pain level.

	AU1	AU2	AU4	AU5	AU6	AU7	AU41	AU42	AU43	AU44	AU45	AU46	AU38	AU39	AU9	AU10	AU11	AU12	AU15	AU16	AU17	AU26
0	3	2	2	2	2	3	1	1	0	1	4	7	2	0	2	2	3	0	3	1	1	1
1	2	5	88	3	44	0	70	9	10	22	10	41	3	2	0	1	7	0	0	4	0	2
2	1	3	41	5	40	2	22	2	11	11	9	37	3	0	6	0	47	0	2	5	5	0
3	4	2	40	16	38	32	20	2	41	13	19	18	2	2	39	3	38	0	9	9	8	4

The likelihood ratio test indicated that the full model significantly improved the fit compared to the null model (χ^2^ = 34.21, df = 15, *P* < 0.001). This suggests that the inclusion of the AUs as predictor variables provides a better fit to the data. Furthermore, the Hosmer–Lemeshow test revealed a nonsignificant result (χ^2^ = 8.45, df = 8, *P* = 0.395), indicating that the observed frequencies of pain levels were in good agreement with the expected frequencies based on the multinomial logistic regression model. These results suggest that the model adequately captures the relationship between the AUs and pain levels in Syrian children, providing a satisfactory fit to the data.

Multinomial logistic regression was performed to analyze the association of FAUs with pain levels. Table 4 shows the coefficient values of each AU with the different four grades of pain (0–3). The test indicated that in children with no pain, none of the FAUs was correlated. However, AU43 (eyes closed) exhibited a negative correlation with pain level (0) (− 0.58). This suggests that the presence of AU43 (eyes closed) reduced the likelihood of experiencing no pain.

**Table 4:** Coefficient values of each AU with genuine pain levels.

	AU1	AU2	AU4	AU5	AU6	AU7	AU41	AU42	AU43	AU44	AU45	AU46	AU38	AU39	AU9	AU10	AU11	AU12	AU15	AU16	AU17	AU26
0	0.05	− 0.02	0.01	0.03	0.06	0.01	0.01	0.03	− 0.58*	0.02	0.09	0.01	0.02	0.01	0.07	0.08	0.09	0.00	0.11	0.04	0.08	0.12
1	0.01	0.01	0.45*	0.02	0.76*	− 0.01	1.21*	0.09	0.08	0.04	0.06	0.99*	0.02	0.01	0.09	0.07	0.08	0.00	0.03	0.07	0.09	0.04
2	0.09	0.07	0.77*	0.01	0.61*	0.01	0.33*	0.01	0.09	0.09	0.09	0.12*	0.03	0.02	0.03	0.08	0.45*	0.00	0.08	0.03	0.06	0.08
3	0.03	0.02	0.66*	0.06	0.98*	0.88*	0.04	0.08	0.35*	0.09	0.09	0.05	0.04	0.02	0.22*	0.07	0.22*	0.00	− 0.04	0.09	0.01	0.02

*Coefficient values that are associated with significance (*P*-value < 0.01).

For mild pain (pain level 1), AU4 (brow lowered), AU6 (cheek raiser), AU41 (lid drop), and AU46 (wink) showed significant associations with positive coefficient values of correlation (*P*-value < 0.01). This indicates that the referred AUs represent highly the facial landmarks of mild pain children.

In moderate pain cases (pain level 2), there was a significant association with AU4 (brow lowered), AU6 (cheek raiser), AU41 (lid drop), AU46 (wink), and AU11 (nasolabial deepener) (*P*-value < 0.01). They were positively associated with moderate pain, suggesting an increased likelihood of experiencing moderate pain in the presence of these AUs.

For severe pain (pain level 3), AU4 (brow lowered), AU6 (cheek raiser), AU7 (lid tightener), AU9 (nose wrinkle), AU11 (nasolabial deepener), and AU43 (eyes closed) demonstrated positive correlations that revealed significant associations. This indicates an increased likelihood of experiencing severe pain when these AUs were present.

Moreover, the Chi-square test was used to compare each AU in predicting pain levels between both boys and girls. When comparing the frequency of these AUs between boys and girls using, no significant differences emerged (*P* > 0.05). These findings suggest that the associations between AUs and pain levels do not significantly differ between boys and girls in the studied population.

In analyzing the fake AUs, Table 5 shows the frequencies of activation of each AU in each pain level.

**Table 5:** Frequencies of activation of each AU in each fake pain level.

	AU1	AU2	AU4	AU5	AU6	AU7	AU41	AU42	AU43	AU44	AU45	AU46	AU38	AU39	AU9	AU10	AU11	AU12	AU15	AU16	AU17	AU26
0	0	2	27	2	2	3	1	1	0	1	4	10	33	0	2	2	3	25	3	1	1	1
1	2	3	75	5	65	2	11	15	97	22	10	22	99	5	2	2	7	75	3	8	3	2
2	8	5	55	8	66	2	12	2	60	7	9	5	50	0	45	0	46	52	2	2	8	2
3	2	2	45	12	41	39	20	2	49	13	14	12	49	2	39	3	37	39	7	9	7	4

Multinomial logistic regression was also performed to analyze the association of FAUs with pain levels while the children were experiencing fake pain. Table 6 shows the coefficient values of each AU with the pain levels. The test showed that in children with no pain, AU4 (brow lowered), AU12 (lip corner puller) and AU38 (nostril dilator) were the most correlated. For fake mild pain, more FAUs were activated than the genuine sensation. AU4 (brow lowered), AU6 (cheek raiser), AU43 (eyes closed), AU38 (nostril dilator), and AU12 (lip corner puller) showed significant associations with positive coefficient values of correlation (*P*-value < 0.01).

**Table 6:** Coefficient values of each AU with genuine pain levels.

	AU1	AU2	AU4	AU5	AU6	AU7	AU41	AU42	AU43	AU44	AU45	AU46	AU38	AU39	AU9	AU10	AU11	AU12	AU15	AU16	AU17	AU26
0	0.04	− 0.02	0.43*	0.03	0.09	0.01	0.01	0.03	0.01	0.02	0.09	0.01	0.55*	0.01	0.07	0.08	0.09	0.29*	0.11	0.05	0.08	0.12
1	0.02	0.01	0.45*	0.02	0.46*	− 0.01	0.09	0.06	0.58*	0.04	0.06	0.01	0.29*	0.01	0.09	0.07	0.08	0.58*	0.03	0.02	0.09	0.04
2	0.02	0.04	0.87*	0.01	0.69*	0.01	0.02	0.01	0.51*	0.09	0.02	0.01	0.45*	0.02	0.81*	0.07	0.42*	0.29*	0.01	0.03	0.03	0.08
3	0.08	0.01	0.46*	0.06	0.88*	0.89*	0.02	0.08	0.39*	0.08	0.06	0.05	0.43*	0.02	0.22*	0.07	0.44*	0.88*	− 0.04	0.06	0.02	0.02

In fake moderate pain, there was a significant association with AU4 (brow lowered), AU6 (cheek raiser), AU43 (eyes closed), AU11 (nasolabial deepener), AU9 (nose wrinkle), AU12 (lip corner puller), and AU38 (nostril dilator) (*P*-value < 0.01).

For fake severe pain, AU4 (brow lowered), AU6 (cheek raiser), AU7 (lid tightener), AU9 (nose wrinkle), AU11 (nasolabial deepener), AU43 (eyes closed), AU12 (lip corner puller) and AU38 (nostril dilator) demonstrated positive correlations that revealed significant associations.

As a result, AU 12 (lip corner puller) and AU 38 (nostril dilator) were the most common AUs that were associated with each fake pain level. On the other hand, AU43 (eyes closing) was associated with each pain level (1–3). This means that when children are mimicking pain sensations, they usually close their eyes. Whereas, in the genuine pain feelings, only children with severe pain activated AU43 (eyes closed).

Moreover, the Chi-square test was also used to compare each AU in predicting fake pain levels between both boys and girls. When comparing the frequency of these AUs between boys and girls using, no significant differences emerged (*P* > 0.05).

## Discussion

The Facial Action Coding System (FACS) is a widely used and validated tool for objectively analyzing facial expressions and decoding emotional and physical states, including pain^21^. It provides a systematic framework for identifying and measuring specific facial muscle movements, known as AUs, which correspond to various facial expressions^22^. By utilizing FACS, researchers can gain valuable insights into pain experiences and assess pain levels objectively, transcending language and cultural barriers^23^.

It is essential to recognize that facial expressions of pain may differ across races and ethnicities^24^. Cultural and individual factors can influence the display and interpretation of pain-related facial expressions^25^. Therefore, it crucial to consider the specific cultural and social context in which the study was conducted when analyzing and interpreting facial expressions of pain^24^.

This study aimed to investigate the association between specific AUs and both genuine and fake pain levels in a sample of Syrian children. Pain assessment and management in children, particularly in dental settings pose significant challenges due to the subjective nature of pain and the limited verbal communication abilities of young children.

The importance of conducting this study within the Syrian community lies in the lack of research available on pain experiences during dental procedures in this population. By focusing on Syrian children, the study contributes to understanding the unique socio-cultural context and potential variations in pain expression and perception within this specific community.

Studying fake AUs provides valuable insights into the complex nature of facial expressions and their role in pain communication^26^. The ability to discern between genuine and fake pain expressions can assist in detecting potential instances of malingering or the exaggeration of pain symptoms, leading to more appropriate treatment interventions, resource allocation, and pain assessment^17^.

The design of this study employed a cross-sectional approach, aiming to investigate the association between specific AUs and pain in a sample of Syrian children during dental injections. The primary objective was to identify the AUs most significantly associated with pain in this specific population.

The study included a targeted age group of children (6–9 years), recognizing that pain experiences may vary across different developmental stages (motor and social development)^27^. By focusing on a specific age range, the study aimed to provide insights into pain responses in a homogenous sample, reducing potential confounding factors related to age differences^28^.

To assess pain levels during dental injections, the FLACC (Face, Legs, Activity, Cry, Consolability) pain assessment scale was utilized. This validated tool enabled objective and standardized pain assessment, capturing multiple dimensions of pain expression and behavior in young children^29^.

To analyze facial expressions accurately, the Facial Action Coding System (FACS) was employed. FACS allowed for the identification and measurement of specific AUs associated with pain, enabling a precise and detailed assessment of facial responses during dental injections^30^. While analyzing the FAUs, lower face AUs that heavily rely on lip and mouth movements were excluded. This aimed to minimize potential confounding factors related to dental interventions involving the lips and mouth, ensuring that the selected AUs specifically captured responses associated with pain experienced during dental injections^31^.

The selection of the inferior alveolar nerve block (IANB) as the dental injection technique provided a standardized and consistent pain-inducing procedure for all participants^32^. This approach facilitated the comparison of pain responses across individuals and allowed for a reliable assessment of the association between AUs and pain levels.

Furthermore, the insertion point of the needle during the IANB procedure served as the pain reference in this study. Utilizing a consistent pain reference across all participants, it enhanced the accuracy and reliability of the pain assessment and minimized potential variations related to different injection sites^33^.

In analyzing fake FAUs, children were asked to mimic pain after the injection rather than before. This offered valuable perspectives on the nature of facial expressions and their relevance to pain assessment. Additionally, this gives us more data about which AUs are the most activated in fake pain.

The results of the study indicated significant associations between specific AUs and pain levels in the study sample. For instance, AU4, AU6, AU41, and AU46 were found to be significantly associated with mild pain. These findings suggest that the activation of these AUs in the upper face may serve as reliable indicators of mild pain experienced during dental injections among Syrian children. Furthermore, the same FAUs were correlated to moderate pain in addition to AU11 (nasolabial deepener). These findings indicate that if AU11 is also activated, moderate pain is suggested.

In severe pain, AU4, AU6, AU7, AU9, AU11 and AU43 were strongly correlated. As a result, lid tighteners, nose wrinkle, and closed eyes are unique indicators of severe pain in Syrian children. Additionally, the presence of AU43 demonstrated a unique negative association with pain absence, suggesting its potential as a protective factor against pain experiences.

Our findings were inconsistence with the Nerella et al. study, which resulted in the correlation of AU25, 26, and 43 with pain. The difference between the results can be justified for many reasons; they focused on the adult population, different races, and hospital patients who are critically ill^34^. While our study aimed to assess pain in children who are receiving IANB.

On the other hand, Facial Action Summary Score was validated in the study of Bringuier et al. and it resulted in the association of AU4, 11, and 43 with pain in children^6^. However, hey did not state this association regarding pain intensity. Moreover, they found that upper lip raiser and lower lip raiser are also correlated. In contrast, our study did not find those AUs as indicators for pain due to the clinical interventional situation we studied the children (during dental injections).

In the study of Prakachin, AU4, 6, 7, 9, 10, 20, 27and 43 were mentioned as the pain indicators. However, they used different pain indicators on adults and children^35^.

Furthermore, our results indicated that specific AUs, such as AU12 and AU38, were predominantly associated with fake pain expressions rather than genuine pain. This finding highlights the importance of distinguishing between genuine and simulated pain when interpreting facial expressions. AU12, which involves the pulling of lip corners, was found in the study of Craig et al. that is more activated in fake pain than in genuine pain^19^. In this regard, the study of Lucey et al. also showed that AU12 is activated in fake pain more frequently^36^.

AU38, which relates to dilating nostrils, may have particular relevance in the context of fake pain as individuals may consciously manipulate these facial movements to portray a sense of discomfort or distress. The nostrils have been observed to dilate or widen during various emotional and physiological states, including, stress^37^.

In this regard, the present study showed that AU4 and AU43 were activated in most of the fake pain expressions of the children. Our findings are in agreement with the study of Ya-Bin Sun et al. in which the authors found that both AU4 and AU12 are involved in mimicking pain expressions^38^.

The study's findings have important implications for pain assessment and management in various clinical settings. By recognizing the differences between real and fake pain expressions, healthcare professionals can enhance their ability to accurately evaluate and respond to patients' pain experiences^39^.

Our study showed no difference between boys and girls regarding the FAUs associated with pain. In the study of Rocha et al., it was also shown that there is no difference between boys and girls regarding pain facial expressions^40^. Additionally, a review (2007) showed that gender does not significantly affect pain experience in children^41^.

The findings of this study will have practical implications for dental professionals, enabling them to recognize and respond to pain more effectively during dental procedures involving injections. Ultimately, this research aims to alleviate pain and enhance the dental experience of Syrian children, fostering positive attitudes toward oral health and reducing dental anxiety in this vulnerable population.

While this study contributes valuable insights into the association between specific AUs and pain levels in Syrian children, it is important to acknowledge certain limitations that should be considered when interpreting the findings. Firstly, the study sample consisted of a specific population of Syrian children, which may limit the generalizability of the results to other populations or cultural contexts. Cultural factors, pain perception, and pain expression can vary across different ethnicities and communities. Therefore, caution should be exercised when extrapolating the findings to broader populations.

Secondly, the study focused on a specific age group of Syrian children, and the findings may not apply to other age ranges or developmental stages. Pain perception and expression can differ among infants, toddlers, and adolescents. Therefore, it is crucial to consider age-related factors when interpreting the results and extrapolating them to different age groups.

Moreover, the study solely examined upper-face AUs while excluding lower face AUs that rely on lips and mouth movements. While this approach aimed to isolate pain-related expressions during dental injections, it may have limited the comprehensive assessment of facial expressions associated with pain. Future studies should consider including lower-face AUs to provide a more comprehensive analysis of pain-related facial expressions.

It is important to mention that this research serves as a crucial foundation for our ongoing study aimed at developing an AI algorithm to detect pain levels in children during dental injections. By investigating the association between facial action units and pain experiences, we gain valuable insights into the complex dynamics of pain expression in Syrian children. Understanding the nuances of facial expressions, both genuine and potentially fake, provides us with essential knowledge that can inform the development of an accurate and reliable AI algorithm.Kindly check and confirm the layout for Tables 1, 3, 4, 5, 6 is correctly identified.they were correctly identified 

## Conclusion

This study employed a well-designed approach, focusing on the unique AUs involved with dental injection pain in Syrian children. It was concluded that AUs 4, 6, 41, and 46 were associated with mild pain, and AUs 4, 6, 41, 46, and 11 were associated with moderate pain cases. In severe pain, AUs 4, 6, 7, 9, 11, and 43 were associated. In fake pain feelings, AU43, AU38, and AU12 were the most activated with no difference between boys and girls.

## Data Availability

The data provided for the results presented in this study is available through the corresponding author upon request.
